# P-1245. Optimizing Antibiotic Dosing in Burn Patients: Real-Time Therapeutic Drug Monitoring for Enhanced Therapeutic Efficacy

**DOI:** 10.1093/ofid/ofae631.1427

**Published:** 2025-01-29

**Authors:** Shon Paulson, Sanju Biju

**Affiliations:** St. Joseph's College of Pharmacy, Thrissur, Kerala, India; St. Joseph's College of Pharmacy, Thrissur, Kerala, India

## Abstract

**Background:**

Burn patients often receive antibiotic therapy, but optimal dosing can be challenging due to variability in pharmacokinetics. This study aimed to assess the accuracy of antibiotic dosing in burns and the impact of real-time dose adjustment via therapeutic drug monitoring (TDM).

**Methods:**

Given the challenges in predicting pharmacokinetics in burn patients, we conducted a 3-year prospective, monocentric, randomized, controlled trial to assess antibiotic dosing accuracy and the effect of systematic therapeutic drug monitoring (TDM) with same-day real-time dose adjustment to maintain therapeutic antibiotic concentrations. Forty-three consecutive burn patients receiving antibiotics were screened at a tertiary care hospital in India. Forty met the inclusion criteria; one declined participation, and another withdrew consent, leaving 21 patients each in the intervention and standard-of-care groups. A total of 38 infection episodes were analysed.

**Results:**

Before the intervention, only 35 out of 67 (52%) initial trough concentrations were within the therapeutic range. No significant difference was found between groups in the initial trough concentrations (adjusted hazard ratio = 1.39 [95% CI, 0.81 to 2.39], P = 0.227) or the time to achieve the target concentration. However, due to real-time dose adjustments, the intervention group showed a higher proportion of trough concentrations within the predefined range (38/51 [74.5%] versus 33/58 [56.9%]; adjusted odds ratio [OR] = 2.34 [95% CI, 1.17 to 4.81], P = 0.018), spent more days within the target range (157 days/245 days on antibiotics [64.1%] versus 138 days/253 days on antibiotics [54.5%]; adjusted OR = 1.64 [95% CI, 1.16 to 2.32], P = 0.005), and had fewer subtherapeutic trough concentrations (19/88 [21.6%] versus 32/99 [32.3%]; adjusted OR = 0.47 [95% CI, 0.26 to 0.87], P = 0.015). No significant difference in infection outcomes was observed between the study groups.

**Conclusion:**

Systematic TDM with same-day real-time dose adjustment was effective in achieving and maintaining therapeutic antibiotic concentrations in burn patients, minimizing both over- and underdosing.

**Disclosures:**

**All Authors**: No reported disclosures

